# Hygeian Home

**Published:** 1878-07-20

**Authors:** 


### MICHIGAN UNIVERSITY.

We invite attention to the advertisement of the above named institution, which will be found in the present number of our journal.

### LACTOPEPTINE.

This preparation, so rapidly coming into professional favor, is advertised in our journal, and is now manufactured by the New York Pharmacal Association. See advertisement in this number.

### HYGEIAN HOME—BEAUTIFUL!

Any medical man, ambitious to establish a health resort, sanitarium, hydropathic institute or hygeian home, can learn of a place admirably adapted to these purposes by addressing the managing editor of this journal. Large dwelling, etc., on the place.

